# TATA Binding Protein (TBP) Promoter Drives Ubiquitous Expression of Marker Transgene in the Adult Sea Anemone *Nematostella vectensis*

**DOI:** 10.3390/genes11091081

**Published:** 2020-09-16

**Authors:** Yael Admoni, Itamar Kozlovski, Magda Lewandowska, Yehu Moran

**Affiliations:** Department of Ecology, Evolution and Behavior, Alexander Silberman Institute of Life Sciences, The Hebrew University of Jerusalem, Jerusalem 9190401, Israel; yael.admoni@mail.huji.ac.il (Y.A.); itamar.kozlovski@mail.huji.ac.il (I.K.); magda.lewandowska@mail.huji.ac.il (M.L.)

**Keywords:** *Nematostella vectensis*, transgenic animal, ubiquitous promoter, gene expression

## Abstract

*Nematostella vectensis* has emerged as one as the most established models of the phylum Cnidaria (sea anemones, corals, hydroids and jellyfish) for studying animal evolution. The availability of a reference genome and the relative ease of culturing and genetically manipulating this organism make it an attractive model for addressing questions regarding the evolution of venom, development, regeneration and other interesting understudied questions. We and others have previously reported the use of tissue-specific promoters for investigating the function of a tissue or a cell type of interest in vivo. However, to our knowledge, genetic regulators at the whole organism level have not been reported yet. Here we report the identification and utilization of a ubiquitous promoter to drive a wide and robust expression of the fluorescent protein mCherry. We generated animals containing a TATA binding protein (TBP) promoter upstream of the mCherry gene. Flow cytometry and fluorescent microscopy revealed expression of mCherry in diverse cell types, accounting for more than 90% of adult animal cells. Furthermore, we detected a stable mCherry expression at different life stages and throughout generations. This tool will expand the existing experimental toolbox to facilitate genetic engineering and functional studies at the whole organism level.

## 1. Introduction

Over the past two decades the sea anemone *Nematostella vectensis* has gained popularity as a model organism for studying molecular evolution [1]. *N. vectensis* is particularly important for comparative biology since it is a representative model species of Cnidaria, which includes sea anemones, corals, hydroids and jellyfish. This phylum is a sister group to Bilateria and diverged approximately 600 million years ago from the vast majority of animal clades [1,2]. Ease of culturing in the laboratory [3], the ability to induce spawning reproducibly to provide thousands of eggs on a weekly basis [4,5], and high genetic homogeneity of the common *Nematostella* lab strain [6], make it an attractive model organism. Interestingly, sequencing of the *Nematostella* genome revealed high complexity of gene content, exon–intron structure, and large-scale gene linkage, which is more similar to vertebrates than to model invertebrate animals like flies or nematodes [7]. This discovery, together with improvements in genome editing technologies, have paved the way for functional studies aiming to provide insights into the origin of complex traits.

We and others have previously reported the use of tissue-specific promoters to study in vivo gene functions, gene regulatory networks and developmental processes in *N. vectensis*. For instance, we probed the dynamics of venom composition in cnidocytes, the cells that deliver toxins in Cnidaria, and demonstrated that venom composition and the population of cells producing that venom change drastically throughout different developmental stages [6]. Other groups used tissue-specific promoters for studying muscle development and neuronal subpopulations [8,9,10,11].

Despite the growing number of tissue-specific transgenic reporter lines, to our knowledge, no ubiquitous promoter that is constitutively expressed in all cell types has ever been reported and extensively characterized in *N. vectensis*. While several transgenic lines generated by others utilized commonly used vertebrate ubiquitous promoters, such as Elongation Factor 1α (EF1α) and actin for stable expression [12,13] and a ubiquitin promoter for transient expression [14], to study development, none of them have been shown to drive a stable and nearly ubiquitous expression, neither at the life cycle level nor at the transgenerational level. Identification of such a ubiquitous and robust promoter will allow an unprecedented ability to perform genetic manipulations such as overexpression, knockdown, knock-in and knockout at the whole organism level. In mice, for instance, the ROSA26 locus, which drives a ubiquitous and constitutive expression in embryos and adults, has been widely utilized for generating hundreds of knock-in mice using the Cre-Lox recombination [15,16,17,18,19]. This allowed expression of a wide array of transgenes including reporters, recombinases, Cas9 and noncoding RNAs. In zebrafish, the ubiquitin promoter was used for ubiquitous transgene expression and Cre-based recombination [20]. In the cnidarian model species *Hydra vulgaris* the β-actin promoter was used to drive a ubiquitous expression of EGFP [21]. The combination of a tissue-specific promoter and a ubiquitous promoter is extremely useful for investigating cell function and to track cell populations in vivo by cell ablation, a technique in which a cell of interest is specifically destroyed by a toxin [22], and lineage tracing, in which a specific cell type is labeled with a fluorescent marker [23]. Both methods typically involve breeding a tissue-specific inducible Cre expressing mouse with a ubiquitously expressing floxed transgene, a toxin, its receptor or a fluorescent marker, that is activated upon Cre-mediated recombination.

The TATA binding protein (TBP) is a subunit of the eukaryotic general transcription factor TFIID, which binds to AT rich elements known as the TATA box found on many eukaryotic promoters [24]. Binding of TFIID to the TATA box is the initial transcriptional step in the formation of the preinitiation complex, which plays an essential role in the activation of eukaryotic genes transcribed by RNA polymerase II [25,26,27]. TBP’s role in basal transcription, its conservation, and histone marks associated with active transcription at its promoter region in different life cycle stages of *N. vectensis* [28], make it a good candidate for a ubiquitous promoter. Importantly, the TBP gene was shown in RT-qPCR to be strikingly stable in its expression across mammalian tissues and to be even more consistent than actin or GAPDH [29]. Indeed, in this study, we demonstrated that the TBP locus is ubiquitous and constitutively active throughout different life stages in *N. vectensis.* We showed that the TBP promoter drives the expression of the fluorescent protein mCherry [30] in a variety of cell types, accounting for about 90% of the cells in primary and adult polyps and 60% of the cells in 4 day old planulae.

## 2. Materials and Methods

### 2.1. Sea Anemone Culture

*Nematostella* embryos, larvae and juveniles were reared in 16‰ sea salt water at 22 °C. Adults were kept in the same salinity but at 18 °C. Polyps were fed with *Artemia salina* nauplii three times a week. Induction of gamete spawning was performed according to a previously described protocol [31].

### 2.2. Cloning

**Figure 1:**
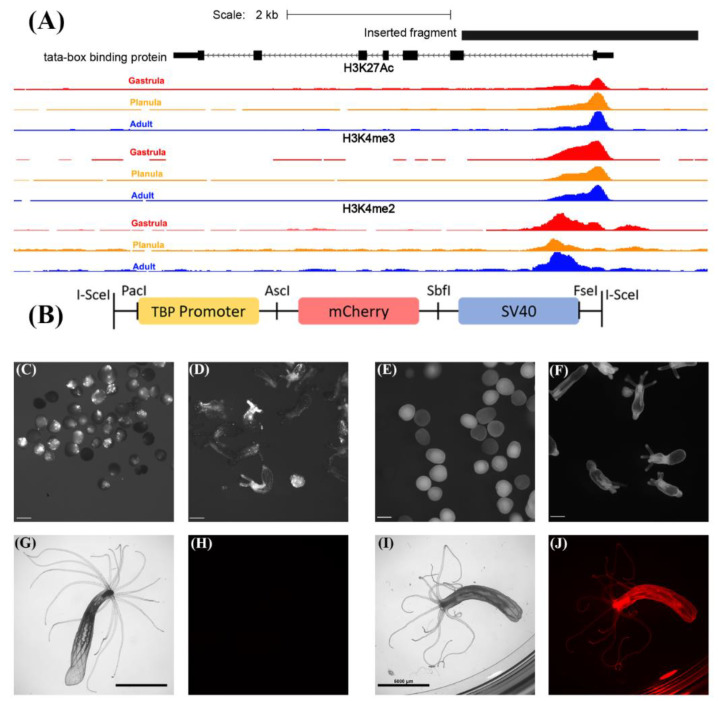
Generation of the TBP::mCherry transgenic line. (**A**) Inserted fragment of the TBP cis element is shown in black. Histone marks associated with active transcription are shown for gastrula (red), planula (orange) and adult (blue). Scale = 2 kb, scaffold_42:923,976-926,863 of *N. vectensis* genome. ChIP-Seq data obtained from [28]. Orientation: right to left. (**B**) Schematic description of the vector used for transgenesis. Adapted from [8]. A 2.9 kb TBPpromoter flanked by *Pac*I and *Asc*I was inserted upstream of the mCherry gene. The SV40 polyadenylation signal and flanking I-*Sce*I meganuclease restriction sites are also depicted. (**C**) F_0_ planulae TBP::mCherry (4 days old) displaying somatic mosaic patches of mCherry expression. Scale bar 0.2 mm. (**D**) F_0_ primary polys (10 days old) displaying somatic mosaic of mCherry expression. Scale bar 0.2 mm. (**E**) F_1_ planulae, including positive transgenes expressing mCherry (shown in white). Scale bar 0.1 mm. (**F**) F_1_ primary polyps (10 days old) expressing mCherry (shown in white). Scale bar 0.2 mm. (**G**) Brightfield image of adult wild-type polyp (about 6 months old), scale bar 5 mm. (**H**) Red channel image of the adult wild-type polyp shown in panel G. Fluorescence in panel H was undetectable using the same exposure parameters as used in panel J. (**I**) Brightfield image of F_1_ heterozygous TBP::mCherry adult. Scale bar 5 mm. (**J**) Red channel image of the individual shown in (**I**).

Approximately 2.9 kb of the TBP promoter including the 5′ UTR, first intron and a small part of the first exon of the TBP gene (Figure 1A and Figure S1, scaffold_42:923,976-926,863 of the *N. vectensis* genome [7]) were PCR amplified with primers containing *Pac*I and *Asc*I restriction sites at the 5′ and the 3′ ends, respectively (Restriction sites are underlined. Forward primer sequence: 5′-TTTTTTAATTAAGCCAGTACACTTCAGTGGGTAC-3′; Reverse primer sequence: 5′-TTTTGGCGCGCCCGCTACCTCCGCTACCTCCAGAAATCAAGCTGCTGTGATG-3′). Following amplification, the resulting DNA fragment was purified by the gel purification kit (Macherey-Nagel, Düren, Germany), digested with the corresponding restriction enzymes (New England Biolabs, Ipswich, MA, USA), and inserted upstream of the mCherry gene in a modified I-*Sce*I site-containing vector as previously described [8,32] (Figure 1B).

### 2.3. Transgenesis

The plasmid was injected into *N. vectensis* zygotes along with the yeast meganuclease I-*Sce*I (New England Biolabs) for efficient integration into the genome as previously described [8,32]. Transgenic animals were visualized under an SMZ18 stereomicroscope equipped with a DS-Qi2 camera (Nikon, Tokyo, Japan). Pictures were edited with Photoshop (ver. 21.2.1; Adobe, Mountain View, CA, USA). All manipulations were performed evenly on photographs and no manipulations were made to enhance unevenly parts of any picture. F_0_ injected individuals were visualized under the stereomicroscope at 3 days old and were further reared according to successful transgenesis. Reproductive adults were induced for gametes at approximately 4 months old and crossed with wild-type (WT) to generate TBP::mCherry heterozygotes. Positive F_1_ individuals were selected and reared. For further analysis, individuals descending from a single F_0_ founder were used.

### 2.4. Cell Dissociation

Animals were dissociated into single cells as previously described [33]. Briefly, adult individuals were immobilized by adding 1/10 volume of 1 M MgCl_2_ into 1/3 strength artificial seawater (17 mM of TrisHCl, 165 mM of NaCl, 3.3 mL of KCl and 9 mM of NaHCO_3_; final solution pH 8.0). Adult polyps were dissected and placed in 1/3 strength calcium/magnesium free and EDTA free artificial seawater. Planulae, primary and dissected adult polyps were washed twice with 1/3 strength artificial seawater and incubated with 50 µg/mL liberaseTM (Roche, Basel, Switzerland) at 37 °C for 10–20 min with occasional pipetting, until fully dissociated. For planulae (4 days old) and primary polyps (10 days old) dissociation, about 200-500 individuals were used per tube. The reaction was stopped by adding 1/10 volume of 500 mM EDTA solution. Cells were centrifuged at 500× *g* at 4 °C and resuspended in 1/3 strength artificial seawater containing 2 µg/mL calcein AM (Enzo, Farmingdale, NY, USA) and 100 nM sytox blue (BioLegend, San Diego, CA, USA) to monitor viability. The suspension was filtered using 5 mL round-bottom tubes with 35 µm cell strainer cap (Corning, NY, USA). Cells were incubated with the viability dyes for 30 min at room temperature and examined by fluorescence microscopy or flow cytometry.

### 2.5. Flow Cytometry

FACSAria III (BD Biosciences, San Jose, CA, USA) equipped with 488-, 405- and 561-nm lasers was used to determine mCherry expression in single cells. Per run, 30,000 events were recorded. Sytox blue positive events were excluded and calceinAM positive events were included. FCS files were further analyzed using FlowJo V10 (BD Biosciences). Each measurement consisted of 3 biological replicates, unless indicated otherwise.

### 2.6. Fluorescence Microscopy

Single cells were visualized with an Eclipse Ni-U microscope equipped with a DS-Ri2 camera and Elements BR software (Nikon). After tissue dissociation, the cells were laid on positively charged glass slides (Bar Naor Ltd., Petah Tikva, Israel). Pictures were edited with Photoshop and all manipulations were performed evenly on photographs and no manipulations were made to enhance unevenly part of any picture.

## 3. Results

### 3.1. Generation of the TBP::mCherry Nematostella vectensis Transgenic Line

Since TBP is a basal transcription factor required for transcription from many eukaryotic promoters [34,35] and since its promoter exhibits histone marks, such as H3K27Ac, H3K4me3 and H3K4me2 (Figure 1A), which are associated with active transcription at the gastrula, planula and adult stages of *N. vectensis* [28], we hypothesized that this promoter will drive the ubiquitous expression of genes at different life cycle stages. To test this hypothesis, we randomly integrated a construct containing a TBP promoter upstream of the mCherry gene into the genome using the well-established yeast I-*Sce*I meganuclease system [8,32] (Figure 1B). Examination of F_0_ microinjected individuals revealed somatic mosaic patches of mCherry expression in planulae and primary polyps as previously reported for other promoters [32] (Figure 1C,D). However, this pattern was eliminated at F_1_, upon breeding with WT individuals_,_ producing an evenly distributed mCherry expression in heterozygous individuals throughout the larval stage into adulthood (Figure 1E,F,I,J). In contrast, WT individuals exhibited negligible red fluorescence concentrated mostly at the pharynx, as previously reported [36]. However, this fluorescence was invisible using the same exposure parameters (Figure 1G,H). Red fluorescence was detectable in TBP::mCherry embryos 30 h post fertilization (Figure S2A,B) and was intensified within 40 h post fertilization (Figure S2C,D). Furthermore, using the NvERTx platform [37], we compared the expression levels of the TBP transcript based on two publicly available datasets [38,39]. While the expression in the first 48 h after fertilization is quite noisy, TBP expression level between 2 and 9 days post fertilization (up to the primary polyp stage) is remarkably stable, as expected of a house-keeping gene (Figure S2E).

### 3.2. TBP Promoter Drives the Expression of mCherry in Diverse Cell Types

**Figure 2:**
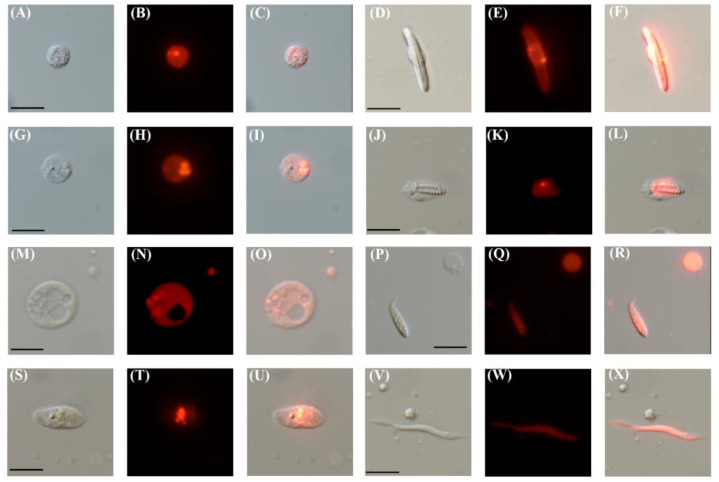
TBP::mCherry *N. vectensis* adults express mCherry in diverse cell types. (**A**) Differential interference contrast (DIC) image of the putative gland cell. (**B**) Red channel of (**A**). (**C**) Merged image of (**A**,**B**). (**D**) DIC image of a nematocyte. (**E**) Red channel of (**D**). (**F**) Merged image of (**D**,**E**). (**G**) DIC image of a putative gland cell. (**H**) Red channel of (**G**). (**I**) Merged image of (**G**,**H**). (**J**) DIC image of a developing spirocyte. (**K**) Red channel of (**J**). (**L**) Merged image of (**J**,**K**). (**M**) DIC image of a putative gland cell (**N**) Red channel of (**M,O**) Merged image of (**M**,**N**). (**P**) DIC image of a spirocyte, bottom left. (**Q**) Red channel of (**P**). (**R**) Merged image of (**P**,**Q**). (**S**) DIC image of a putative gland cell (**T**) Red channel of (**S**). (**U**) Merged image of (**S**,**T**). (**V**) DIC image of a muscle cell. (**W**) Red channel of (**V**). (**X**) Merge image of (**V**,**W**). Scale bars are 10 µm for all images.

Next, we dissociated TBP::mCherry F_1_ polyps into single cells to qualitatively determine the extent and types of mCherry expressing cells. Indeed, we found that the vast majority of cells are mCherry positive accounting for diverse cell types (Figure 2). These included putative gland cells with a typical circular morphology and containing noticeable vesicles of various sizes [6,40,41] (Figure 2A–C,G–I,M–O,S–U), specialized stinging cells such as spirocytes (Figure 2J–L,P–R) and nematocytes (Figure 2D–F), and a muscle cell (Figure 2V–X), identified based on the morphology [42].

### 3.3. TBP::mCherry Is Expressed Stably at Different Life Stages and Throughout Generations in a High Percentage of Cells

**Figure 3:**
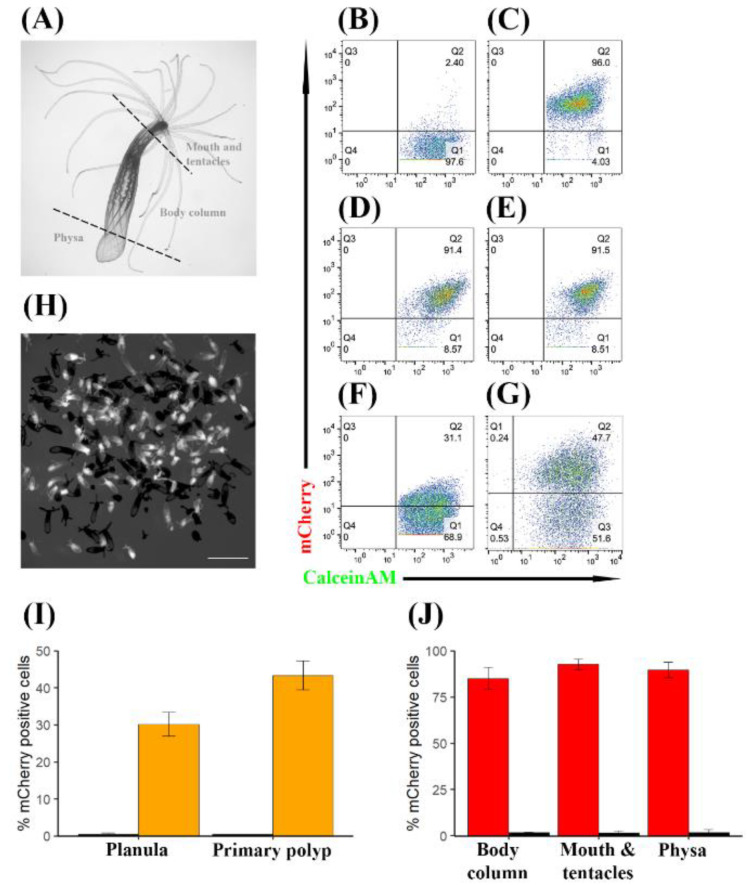
TBP::mCherry *N. vectensis* individuals express mCherry in a high percentage of their cells at different life cycle stages and throughout generations. (**A**) Schematic description of a dissection procedure of an adult polyp. (**B**–**G**) Flow cytometry analysis. mCherry expression is on the Y axis and calceinAM, which served as a viability dye is on the X axis. (**B**) Representative flow cytometry result of dissociated mouth and tentacles obtained from a WT adult. (**C**) Representative flow cytometry result of dissociated mouth and tentacles obtained from the F_1_ TBP::mCherry adult. (**D**) Representative flow cytometry result of dissociated body column obtained from the F_1_ TBP::mCherry adult. (**E**) Representative flow cytometry result of dissociated physa obtained from the F_1_ TBP::mCherry adult. (**F**) Representative flow cytometry result of dissociated F_2_ TBP::mCherry planulae (4 days old). (**G**) Representative flow cytometry result of dissociated F_2_ TBP::mCherry primary polyps. (**H**) Representative image of F_2_ primary polyps used in (**G**). mCherry positive individuals are shown in white and mCherry negative are in black. 95 mCherry negative individuals and 96 mCherry positive individuals were counted in this representative experiment. (**I**) Average percentage of mCherry positive cells in F_2_ TBP::mCherry planulae and primary polyps (orange bars) against their WT counterparts (black bars). Standard deviation is plotted as error bars. (**J**) Average percentage of mCherry positive cells in F_1_ adult polyps (red bars) against their WT counterparts (black bars). Different body parts are shown on the X axis. Standard deviation is plotted as error bars.

In order to quantify the percentage of mCherry positive cells in adult polyps, we performed dissections into three parts: mouth and tentacles, body column and physa [4] (Figure 3A). Importantly, the pharynx, which was reported to exhibit natural red fluorescence, was removed during the dissection. These body parts were dissociated into single cells and stained with the viability dyes sytox blue, a nucleic acid stain that positively marks dead cells due to compromised plasma membrane, and calceinAM, which indicates vital enzymatic activity when positive. The stained cells were subjected to flow cytometry analysis. WT individuals were used to define mCherry positivity. In agreement with a previous report, WT individuals demonstrated a small population of naturally fluorescent cells [36] (Figure 3B,J, Figure S3A,B), accounting for 1.55% (SD = 1.1, *n* = 3), 1.75% (SD = 0.25, *n* = 4), and 1.66% (SD = 1.79, *n* = 3), at the mouth and tentacles, body column, and physa, respectively. On average, TBP::mCherry individuals contained a population of about 90% mCherry positive cells. Specifically, 92.6% (SD = 2.97, *n* = 3) at the mouth and tentacles (Figure 3C,J), 85.1% at the body column (SD = 6.02, *n* = 4; Figure 3D,J) and 89.7% (SD = 4.22, *n* = 3) at the physa (Figure 3E,J).

Next, we sought to determine whether mCherry expression is stable throughout different stages of the life cycle and throughout generations. To this end, we crossed F_1_ TBP::mCherry males with WT females and measured red fluorescence of planulae at day 4 post fertilization and primary polyps at day 10 post fertilization by flow cytometry. We used transgenic males but not transgenic females because the eggs of the latter are loaded with mCherry via maternal protein deposition (Figure S4), hence masking zygotic mCherry expression. Assuming Mendelian inheritance, we expected 50% of the offspring to be WT/WT and 50% TBP::mCherry/WT. Indeed, fluorescence microscopy revealed a roughly even distribution of mCherry positive individuals and mCherry negative individuals (Figure 3H). Flow cytometry analysis of this evenly mixed F_2_ population of individuals revealed 30.13% (SD = 3.22, *n* = 6) mCherry positive cells at the planula stage (Figure 3F,I) and 43.33% (SD = 3.90, *n* = 3) at the primary polyp stage (Figure 3G,I), indicating that mCherry expression increases after metamorphosis and stabilizes at 85–95% in adulthood.

## 4. Discussion

The identification of ubiquitous promoters such as ROSA26 in mice has revolutionized our ability to generate informative mouse models [15,16,17,18]. The emergence of *N. vectensis* as a model organism asks for the development of similar technologies for genetic engineering. The lack of an identified ubiquitous promoter has hampered our ability to perform genetic manipulations required for functional studies of certain model organisms.

In this work, we identified the TBP promoter as a ubiquitous and robust means for stably expressing proteins at the whole organism level throughout different life cycle stages and generations. Specifically, we demonstrated that, despite differences in cell composition [33,43], the TBP promoter drives the expression of mCherry homogeneously across different body parts in adults (Figure 3C–E,J), and that both primary polyps and adults express mCherry in about 85–90% of their cells (Figure 3I,J).This percentage is similar to that reported in bilaterian model organisms using other ubiquitous promoters, as determined by flow cytometry [20,44].

However, it should be noted that each system has its limitations. These limitations, in turn, must be taken into account in order to properly address biological questions by each system. For instance, flow cytometry analysis of ROSA26-EGFP mice [44] demonstrated a tissue dependent EGFP expression pattern. While liver, kidney and heart tissues expressed EGFP in a smaller proportion of cells, ranging from 17% to 64%, other tissues exhibited a greater proportion of EGFP positive cells, accounting for more than 80% of the examined tissues [45]. In zebrafish, the *ubi* promoter exhibited variability in the percent of positive cells, ranging from about 81% to 95%, depending on the examined population of blood cells [20]. Therefore, further investigation should focus on identifying the population of mCherry negative cells in our transgenic line and determining why they are negative. It is possible that in certain cell types the TBP promoter does not sustain mCherry expression due to position effects and silencing. Such effects are expected for randomly integrated transgene constructs, which are susceptible to environmental influences, such as the enhancer/repressor–promoter interaction, heterochromatin-induced gene inactivation and DNA methylation [46,47,48,49].

In this study, we examined a mixed population of heterozygote TBP::mCherry males bred with WT females to estimate mCherry expression in F_2_. In the future, the procedures described in our study could be repeated with inbred homozygotes to obtain a direct estimate of the number of positive cells. However, in light of the clear Mendelian segregation of the transgene (Figure 3H) our estimations would most probably stay the same. Importantly, any future experimental design employing the TBP promoter in *Nematostella* should take into account maternal deposition (Figure S4) as this can dramatically affect the results. Lastly, in the planula stage, in contrast to primary polyps and adults, we observed a significantly lower percent of mCherry positive cells, 60% versus 86% and 90% on average, respectively. Further research is required to explain this difference. It is plausible that this discrepancy is due to differences in cell composition. Indeed, single-cell RNA sequencing analysis of planula versus adult individuals demonstrated a reduced diversity of about 1/3 of the total metacell count in the planula compared to an adult and a distinct population of undifferentiated cells that were only existent in the planula [33]. Alternatively, it is also possible that the accumulation of mCherry above the natural fluorescence threshold in the planula was not yet achieved within 4 days post fertilization. Notably, primary polyps at 10 days post fertilization exhibited a similar percentage of positive cells to that obtained by adults.

Potential applications of the tool presented here will include cell transplantation experiments, generalized overexpression of proteins, and developing tools to probe cell lineages and cell function in a similar manner to the commonly used Cre-lox mice models. To our knowledge, no inducible system, aside of a heat shock promoter [50], has ever been reported in *N. vectensis.* Therefore, future efforts will be focused on developing such a system. Recent advancements in the field of optogenetics [51,52], which uses light to control cell function, and in vivo imaging [53,54,55], have provided a greater resolution to examine biological processes in space and time. The study of the translucent *N. vectensis* can potentially benefit from such technologies. The TBP promoter lays the foundation for the construction of more sophisticated CRISPR-Cas9, recombinase-dependent, drug or light inducible transgene systems for all developmental stages and a wide array of cell types in *N. vectensis.*

## Supplementary Materials

The following are available online at https://www.mdpi.com/2073-4425/11/9/1081/s1, Figure S1: Sequence of the cloned TBP promoter. Figure S2: Early expression of the TBP::mCherry transgene and native TBP gene. Figure S3: Representative flow cytometry analyses of WT *N. vectensis* adult individuals. Figure S4: Maternal deposition of mCherry in the eggs of TBP::mCherry transgenic females.

## References

[1] Layden M.J., Rentzsch F., Röttinger E. (2016). The rise of the starlet sea anemone *Nematostella vectensis* as a model system to investigate development and regeneration. Wiley Interdiscip. Rev. Dev. Biol..

[2] Technau U., Steele R.E. (2011). Evolutionary crossroads in developmental biology: Cnidaria. Development.

[3] Hand C., Uhlinger K.R. (1992). The culture, sexual and asexual reproduction, and growth of the sea Anemone *Nematostella vectensis*. Biol. Bull..

[4] Stefanik D.J., Friedman L.E., Finnerty J.R. (2013). Collecting, rearing, spawning and inducing regeneration of the starlet sea anemone, *Nematostella vectensis*. Nat. Protoc..

[5] Fritzenwanker J.H., Technau U. (2002). Induction of gametogenesis in the basal cnidarian *Nematostella vectensis* (Anthozoa). Dev. Genes Evol..

[6] Columbus-Shenkar Y.Y., Sachkova M.Y., Macrander J., Fridrich A., Modepalli V., Reitzel A.M., Sunagar K., Moran Y. (2018). Dynamics of venom composition across a complex life cycle. eLife.

[7] Putnam N.H., Srivastava M., Hellsten U., Dirks B., Chapman J., Salamov A., Terry A., Shapiro H., Lindquist E., Kapitonov V.V. (2007). Sea anemone genome reveals ancestral eumetazoan gene repertoire and genomic organization. Science.

[8] Renfer E., Amon-Hassenzahl A., Steinmetz P.R.H., Technau U. (2010). A muscle-specific transgenic reporter line of the sea anemone, *Nematostella vectensis*. Proc. Natl. Acad. Sci. USA.

[9] Nakanishi N., Renfer E., Technau U., Rentzsch F. (2012). Nervous systems of the sea anemone *Nematostella vectensis* are generated by ectoderm and endoderm and shaped by distinct mechanisms. Development.

[10] Layden M.J., Johnston H., Amiel A.R., Havrilak J., Steinworth B., Chock T., Röttinger E., Martindale M.Q. (2016). MAPK signaling is necessary for neurogenesis in *Nematostella vectensis*. BMC Biol..

[11] Richards G.S., Rentzsch F. (2014). Transgenic analysis of a SoxB gene reveals neural progenitor cells in the cnidarian *Nematostella vectensis*. Development.

[12] He S., Del Viso F., Chen C.Y., Ikmi A., Kroesen A.E., Gibson M.C. (2018). An axial Hox code controls tissue segmentation and body patterning in *Nematostella vectensis*. Science.

[13] Steinmetz P.R.H., Aman A., Kraus J.E.M., Technau U. (2017). Gut-Like ectodermal tissue in a sea anemone challenges germ layer homology. Nat. Ecol. Evol..

[14] Fritz A.E., Ikmi A., Seidel C., Paulson A., Gibson M.C. (2013). Mechanisms of tentacle morphogenesis in the sea anemone *Nematostella vectensis*. Development.

[15] Casola S. (2010). Mouse models for miRNA expression: The ROSA26 locus. Methods Mol. Biol..

[16] Soriano P. (1999). Generalized lacZ expression with the ROSA26 Cre reporter strain. Nat. Genet..

[17] Kisseberth W.C., Brettingen N.T., Lohse J.K., Sandgren E.P. (1999). Ubiquitous expression of marker transgenes in mice and rats. Dev. Biol..

[18] Srinivas S., Watanabe T., Lin C.S., William C.M., Tanabe Y., Jessell T.M., Costantini F. (2001). Cre reporter strains produced by targeted insertion of EYFP and ECFP into the ROSA26 locus. BMC Dev. Biol..

[19] Platt R.J., Chen S., Zhou Y., Yim M.J., Swiech L., Kempton H.R., Dahlman J.E., Parnas O., Eisenhaure T.M., Jovanovic M. (2014). CRISPR-Cas9 knockin mice for genome editing and cancer modeling. Cell.

[20] Mosimann C., Kaufman C.K., Li P., Pugach E.K., Tamplin O.J., Zon L.I. (2011). Ubiquitous transgene expression and Cre-based recombination driven by the ubiquitin promoter in zebrafish. Development.

[21] Wittlieb J., Khalturin K., Lohmann J.U., Anton-Erxleben F., Bosch T.C.G. (2006). Transgenic Hydra allow In Vivo tracking of individual stem cells during morphogenesis. Proc. Natl. Acad. Sci. USA.

[22] Brockschnieder D., Pechmann Y., Sonnenberg-Riethmacher E., Riethmacher D. (2006). An improved mouse line for Cre-induced cell ablation due to diphtheria toxin A, expressed from the Rosa26 locus. Genesis.

[23] Matthews B.G., Ono N., Kalajzic I. (2019). Methods in lineage tracing. Principles of Bone Biology.

[24] Lee T.I., Young R.A. (2000). Transcription of Eukaryotic Protein-Coding Genes. Annu. Rev. Genet..

[25] Hoffmann A., Sinn E., Yamamoto T., Wang J., Roy A., Horikoshi M., Roeder R.G. (1990). Highly conserved core domain and unique N terminus with presumptive regulatory motifs in a human TATA factor (TFIID). Nature.

[26] Peterson M.G., Tanese N., Pugh B.F., Tjian R. (1990). Functional domains and upstream activation properties of cloned human TATA binding protein. Science.

[27] He Y., Yan C., Fang J., Inouye C., Tjian R., Ivanov I., Nogales E. (2016). Near-Atomic resolution visualization of human transcription promoter opening. Nature.

[28] Schwaiger M., Schönauer A., Rendeiro A.F., Pribitzer C., Schauer A., Gilles A.F., Schinko J.B., Renfer E., Fredman D., Technau U. (2014). Evolutionary conservation of the eumetazoan gene regulatory landscape. Genome Res..

[29] Radonić A., Thulke S., Mackay I.M., Landt O., Siegert W., Nitsche A. (2004). Guideline to reference gene selection for quantitative real-time PCR. Biochem. Biophys. Res. Commun..

[30] Shaner N.C., Campbell R.E., Steinbach P.A., Giepmans B.N.G., Palmer A.E., Tsien R.Y. (2004). Improved monomeric red, orange and yellow fluorescent proteins derived from Discosoma sp. red fluorescent protein. Nat. Biotechnol..

[31] Genikhovieh G., Technau U. (2009). Induction of spawning in the starlet sea anemone nematostella vectensis, In Vitro fertilization of gametes, and dejellying of zygotes. Cold Spring Harb. Protoc..

[32] Renfer E., Technau U. (2017). Meganuclease-Assisted generation of stable transgenics in the sea anemone *Nematostella vectensis*. Nat. Protoc..

[33] Sebé-Pedrós A., Saudemont B., Chomsky E., Plessier F., Mailhé M.P., Renno J., Loe-Mie Y., Lifshitz A., Mukamel Z., Schmutz S. (2018). Cnidarian cell type diversity and regulation revealed by whole-organism single-cell RNA-Seq. Cell.

[34] Imbalzano A.N., Kwon H., Green M.R., Kingston R.E. (1994). Facilitated binding of TATA-binding protein to nucleosomal DNA. Nature.

[35] Antonova S.V., Boeren J., Timmers H.T.M., Snel B. (2019). Epigenetics and transcription regulation during eukaryotic diversification: The saga of TFIID. Genes Dev..

[36] Ikmi A., Gibson M.C. (2010). Identification and In Vivo characterization of NvFP-7R, a developmentally regulated red fluorescent protein of *Nematostella vectensis*. PLoS ONE.

[37] Warner J.F., Guerlais V., Amiel A.R., Johnston H., Nedoncelle K., Röttinger E. (2018). NvERTx: A gene expression database to compare embryogenesis and regeneration in the sea anemone *Nematostella vectensis*. Development.

[38] Helm R.R., Siebert S., Tulin S., Smith J., Dunn C.W. (2013). Characterization of differential transcript abundance through time during *Nematostella vectensis* development. BMC Genom..

[39] Fischer A.H.L., Mozzherin D., Eren A.M., Lans K.D., Wilson N., Cosentino C., Smith J. (2014). SeaBase: A multispecies transcriptomic resource and platform for gene network inference. Integr. Comp. Biol..

[40] Babonis L.S., Ryan J.F., Enjolras C., Martindale M.Q. (2019). Genomic analysis of the tryptome reveals molecular mechanisms of gland cell evolution. EvoDevo.

[41] Frank P., Bleakney J.S. (1976). Histology and sexual reproduction of the anemone *Nematostella vectensis* stephenson 1935. J. Nat. Hist..

[42] Jahnel S.M., Walzl M., Technau U. (2014). Development and epithelial organisation of muscle cells in the sea anemone *Nematostella vectensis*. Front. Zool..

[43] Zenkert C., Takahashi T., Diesner M.O., Özbek S. (2011). Morphological and molecular analysis of the *Nematostella vectensis* cnidom. PLoS ONE.

[44] Giel-Moloney M., Krause D.S., Chen G., Van Etten R.A., Leiter A.B. (2007). Ubiquitous and uniform In Vivo fluorescence in ROSA26-EGFP BAC transgenic mice. Genesis.

[45] Fujiki Y., Tao K., Bianchi D.W., Giel-Moloney M., Leiter A.B., Johnson K.L. (2008). Quantification of green fluorescent protein by in vivo imaging, PCR, and flow cytometry: Comparison of transgenic strains and relevance for fetal cell microchimerism. Cytom. Part A.

[46] Bestor T.H. (2000). Gene silencing as a threat to the success of gene therapy. J. Clin. Investig..

[47] Martin D.I., Whitelaw E. (1996). The vagaries of variegating transgenes. Bioessays.

[48] Kioussis D., Festenstein R. (1997). Locus control regions: Overcoming heterochromatin-induced gene inactivation in mammals. Curr. Opin. Genet. Dev..

[49] Sadelain M., Papapetrou E.P., Bushman F.D. (2011). Safe harbours for the integration of new DNA in the human genome. Nat. Rev. Cancer.

[50] Ikmi A., McKinney S.A., Delventhal K.M., Gibson M.C. (2014). TALEN and CRISPR/Cas9-mediated genome editing in the early-branching metazoan *Nematostella vectensis*. Nat. Commun..

[51] Pastrana E. (2011). Optogenetics: Controlling cell function with light. Nat. Methods.

[52] Deisseroth K., Feng G., Majewska A.K., Miesenböck G., Ting A., Schnitzer M.J. (2006). Next-Generation optical technologies for illuminating genetically targeted brain circuits. J. Neurosci..

[53] Marvin J.S., Shimoda Y., Magloire V., Leite M., Kawashima T., Jensen T.P., Kolb I., Knott E.L., Novak O., Podgorski K. (2019). A genetically encoded fluorescent sensor for in vivo imaging of GABA. Nat. Methods.

[54] Portugues R., Severi K.E., Wyart C., Ahrens M.B. (2013). Optogenetics in a transparent animal: Circuit function in the larval zebrafish. Curr. Opin. Neurobiol..

[55] Yang W., Yuste R. (2017). In Vivo imaging of neural activity. Nat. Methods.

