# Supplementary material for: TATA Binding Protein (TBP) Promoter Drives Ubiquitous Expression of Marker Transgene in the Adult Sea Anemone Nematostella vectensis
Source: Genes (Basel). 2020 Sep 16;11(9):1081. doi: 10.3390/genes11091081 (PMC7565589; doi:10.3390/genes11091081)
Supplement: Supplementary file 1 [file genes-11-01081-s001.pdf]

This file contains supplementary figures S1-S4

Supplementary figure S1: *TBP* Promoter sequence (2907 bp) spanned by *Pac*I and *Asc*I restriction sites:

TTAATTAAGCCAGTACACTTCAGTGGGTACATATTTTTCAATAACTATAAAAATAACAGTTATTTAAAAAAAGAAAA
AAATAATTATCCTCATATGGCATTAGTTATCATTCATATTCATTTTTGCACATTTCGCCATGCACTTTGCCACACGC
TGATACATGGAATTGCTTGCTACAGCAAATTAGCTAATTGGGTACAAAAAGCATCAATTAGGATTCTAAGAAATTCT
AAGCAAAAGTTTTTATCCCTGTCATATTTTTTATATCACGATAAGTAAAGCAGATTAAGATTTGTATGTCATCATAT
TAAAAAAAACTTCAGACTTAACTCTTCACACATCAGAGTTTTTACAAGGATTTAGCAAATCTTTTAGATGGGATTGC
AGATTCCCTAGCAAATTTCGTGATATGGATGTTAGGCATATTGGGGGGGGGGGGAGGTTATTTACAGATATAAGCAG
TTTATATCATACAGGGAGTAAAAATCCTCCCAGAACCATAATAAAACAAATTTTCAGTGATATTGTTGTGGCAGGTA
GTATACCGTTTCTGTTGCCTCCTAACATTTTGTTGGCTTTTAACATCATTCCCCTTCTTCATTGGATTCTATAAAAG
CAATATGTTCTAGTCTTCCTTGCAGTAGTTCTGGACTTGTCATACTAGGTGGCGCTCAAGCACCGAGACTAAATATG
TTCAGGCAGTTCAGTTCATATTTATCCTCTTCGGCAAGGCAGTTATTAGAGAGACAGGCTTATTCAAGCAATTATGG
TATTTTCAGCTATTTGAATATAAAAACACATTGCTACCTGTCAGGTTGCAAAGCCTTAGCTATTGAACAAGTAATAG
TCACCCTAGCAATATTTGCTGGAATGTAAGAAGAAAAAGGTTTAGCCATTTTTTGAATAATGAAAAGACTATCCTGA
GATTTTGTGTTGTTATTGATATATCATAATAATTATAATTCACCTAAGAAAGACTGTACTAAAATTTTCTTTTGATT
TTTTCGACTGTCAGAAACTCTATATTCTTATCTCTATTTCTCTTGCGTGGGCTCACTGTGGCGTTCCCTTGGATCGC
GGGCTCGTGTTAACCCAGCATGCATTTCGCGGCATCTAGTTCAGATTCTATTTGCCGTATGCCGTACATCGTTTGAA
ACGAAAATCAGTAGAAAATAAGAAGAAAAAGGTGAATTCTATCTGCGCTACTCAACGAGGAACCTTCCTCGTGAGTC
CTAGGCGCCAGCAATGGAGCACGAGGATGAAAACCGCTTCAGAAGTTCAAATGTGGTGCAGGTTAGTTCGAGCGTCA
ATGTTGTTCATAATGGAATGGCAGGCTAAATATCAATGACTTTTTAGCAGAACATTATTAATTTTTTGGCTTTCAAT
AGACTGAATAAAGAGACCCATTGTCTTTTCTTTTCATTTGAGTTTTGTTAGGAATAAAAAATATCGAAATAACACCA
TCATTTCTTAGGTTTTTATGTTCTTAATTGTACTGAGATAAAATGTTGCCTCATTCCTCTTGAGAAACAATTTAACA
CGAGAAAATATAGTATAGTTATCAGCAAAGTATAAACTTCTTTTTAAAAATGCAAGATATTGTGTCTTCGCATGAAA
GATCTATACTTAAAATATTTTGCTAGAAGGGTGATAACTCTAGCAGTTAACGATGTAAAACTTGTCATGAACTACTC
AAACCAGATTACACTAATTTCAGTCACATGTGTACCCTGTGCCTTTAGTCCCGACTGTGTTTTGGTGGACATCAATA
ATAACTTTAACATTTTTCCCATCCGCCCTGTTTCTTATCTTTGTGTTGTTGATTTAGCGAGTATGTTTGTATTTATC
TGTTGTCTGCTTTCTTATCTTTGTGTTGTTGATTTAGTGAGTATGTTTGTATTTATCTGTGGTCTGCTTTCCTAAGG
CCTGTCACTTCTTGGCGCTCTCTCATTGGCTAGCTATCCAATAGCTTCCATAACTGGCTTTACAATGAAGTTCTTTA
TTCAGCCGAATGACTAAAATAAAAGGCCCACATAATAAAAATTAACCGTTTAGAATTAACATTACTGGGATTAAAAT
GGTTAATTGAGAACGGGATAATCAAATCAATCCTTTTCGCGGCTTTATATCTGCAGTCAGACCAAAAACACATTATG
GGCGCAGGGTGATACGGAACTATTCCCACGTCCTCCGGCAAGCACGAAAAATTAAAAGCAATGTAAATCGAAAAAAT
TGCATCAAGTCAGAAAAAAAACACACATTAACAGACATTCTTTCTTCTAAGCATTTGTATAAAAATTAGGACATTTT
TGGTAAAGGAGGTATTGTTTTCATCTGACTTTAGTGATGCGGGTCAGTTCACTGAGCAGTAAACACCGTTCATTTTA
GCAACAATTATGAAAATCACTTTGAAATAATGATACTTTCTGAACAAACCATTGGATGAAATAAGTAATTTTGGATT
GTTCAAATCTACAATATTCAAGGCAAAAAGATAAAACAAATGTTGAACACATAACTAGAACTGCTACGAAACTGGTT
ATTGTACTGTACGTGAGATATCTTAAAAACTTGATCTGACAATATTACGCTAAGGGATTTGATCTAGTTTGAACATA
TTAACATGGAGCATGCAGTGTTTGCTATCTATAAATACAACCCCCCTCATTTGGCTTTAGTCTATTTTGCTCTGACG
GACTAACACGATACTACTGAGAGACATAAAACATACCATTCTAGCCAGATTTTGATTTATTTCCTTGATTCCATACC
AATACAGACAACCTAGTTATTGTACTGTACATCTTGTCTGTAGTGTTTCTATTCTATGTAGTATCACTAGTCTTTTG
CGTAATGCTCCAAACTTGTTGTTTAGCATCACAGCAGCTTGATTTCTGGAGGTAGCGGAGGTAGCG**GGCGCGCC**

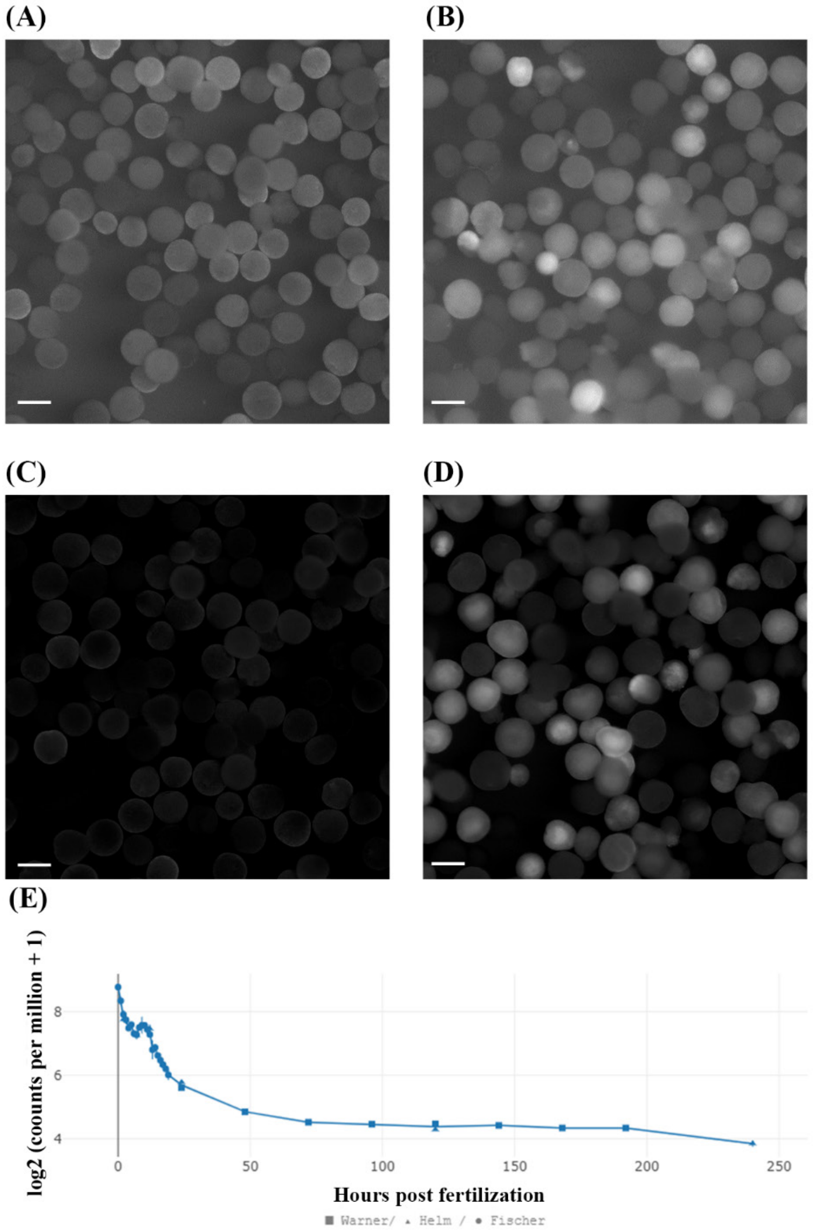

Supplementary figure S2. Early expression of the TBP:mCherry transgene and native TBP gene. TBP:mCherry embryos express mCherry as early as 30 hours post fertilization. **(A)** WT embryos 30 hours after fertilization. **(B)** F_2 WT/TBP:mCherry embryos 30 hours after fertilization. **(C)** WT embryos 40 hours after fertilization. **(D)** F_2 WT/TBP:mCherry embryos 40 hours after fertilization. Red fluorescence is shown in white. Scale bar 0.2mm. Eggs in panels A and B were photographed at a four-fold larger digital gain compared to panels C and D. **(E)** TBP expression levels obtained from quantitative RNA-seq data at the NvERTx platform (Warner et al. 2017 Development 145:dev162867).

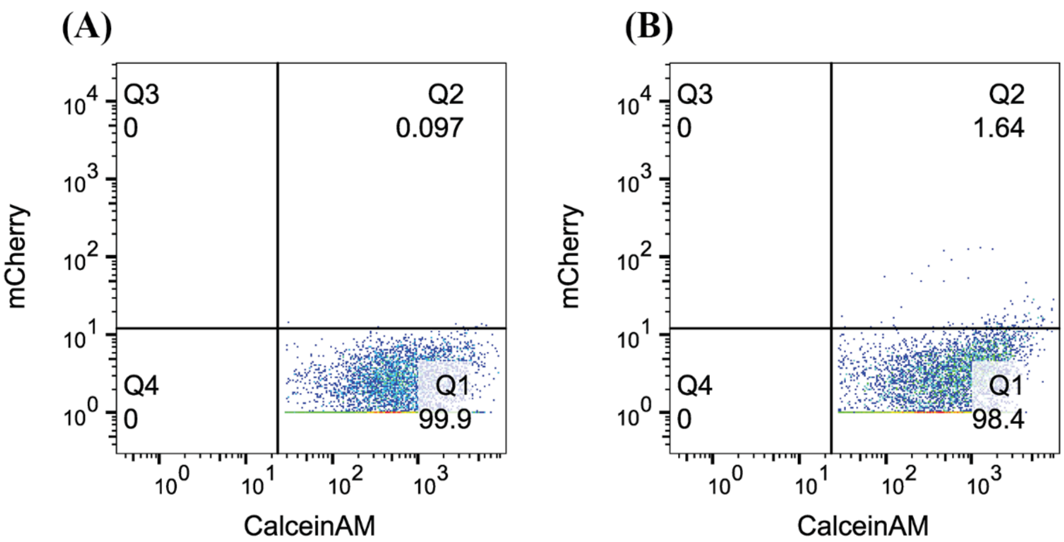

Supplementary figure S3. Representative FACS analyses of WT *N. vectensis* adult individuals. **(A)** FACS analysis of dissociated cells obtained from the physa. **(B)** FACS analysis of dissociated cells obtained from the body column.

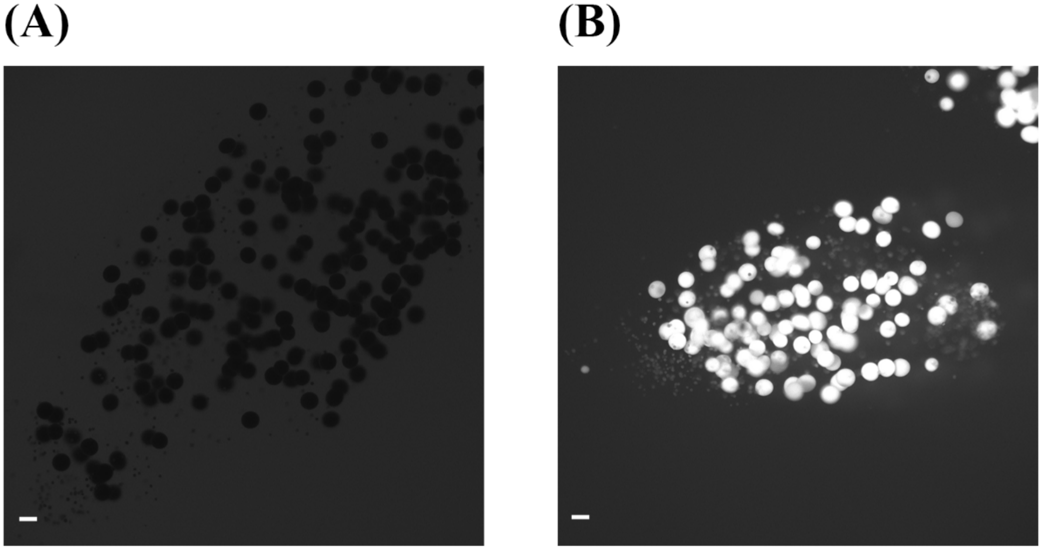

Supplementary figure S4. Maternal deposition of mCherry. **(A)** unfertilized egg package obtained from wildtype females following induction (**B)** unfertilized egg package exhibiting red fluorescence obtained from TBP:mCherry F_1 females following induction. The pictures were taken using the same exposure parameters. Scale bar 0.2 mm.
